# AUDiaL: A Natural Language Interface to Make Statistical Charts Accessible to Blind Persons

**DOI:** 10.1007/978-3-030-58796-3_44

**Published:** 2020-08-10

**Authors:** Tomas Murillo-Morales, Klaus Miesenberger

**Affiliations:** 8 Institute Integriert Studieren, JKU Linz, Linz, Austria; 9 Jack Baskin School of Engineering, UC Santa Cruz, Santa Cruz, CA USA; 10 Dipartimento di Meccanica, Politecnico di Milano, Milan, Italy; 11 Support Centre for Students with Special Needs, Masaryk University Brno, Brno, Czech Republic; Institute Integriert Studieren, Johannes Kepler University, Altenbergerstraße 69, 4040 Linz, Austria

**Keywords:** Natural language interface, Statistical charts, Accessibility, Annotation

## Abstract

This paper discusses the design and evaluation of AUDiaL (Accessible Universal Diagrams through Language). AUDiaL is a web-based, accessible natural language interface (NLI) prototype that allows blind persons to access statistical charts, such as bar and line charts, by means of free-formed analytical and navigational queries expressed in natural language. Initial evaluation shows that NLIs are an innovative, promising approach to accessibility of knowledge representation graphics, since, as opposed to traditional approaches, they do not require of additional software/hardware nor user training while allowing users to carry out most tasks commonly supported by data visualization techniques in an efficient, natural manner.

## Introduction

In order to foster the inclusion of blind and visually impaired persons in the information society it is paramount that they have full access to information in all its forms, a sizeable amount of which is based on or supported by visual means. Graphically displayed information in the form of statistical charts, networks, and maps (which, as a whole, we will refer to as *diagrams*), is commonly employed in newspapers, didactic materials, finance, and many other aspects of daily life. Diagrams exploit the natural perceptual, cognitive, and memorial capacities of human beings so that the represented information can be more easily processed and understood by sighted readers. However, blind persons are generally excluded from accessing diagrammatic representations of data. Diagrams and other graphics have been labelled as “the last frontier in accessibility” [7], since current alternative accessible versions thereof are either not functionally equivalent [8] (e.g. tabular descriptions, sonified diagrams), they have to be greatly simplified and must be authored by a sighted expert using specialized devices (tactile graphics), or some substantial training must be undertaken by the end user (interactive software) before becoming accustomed to their peculiarities so that they can be used efficiently.

In this paper, we expand on our previous concept of a non-visual accessible web interface to diagrams [12] by introducing AUDiaL (Accessible Universal Diagrams through Language), a prototype of a Web-based NLI to semantically-enhanced statistical charts adapted to the specific needs of blind users. NLIs are an innovative means for non-visually accessing diagrams that possess many benefits in comparison with traditional accessible diagrams, namely:

- NLIs leverage a skill that is mastered early in life, natural language, which makes them a useful and efficient way for people to interact with computers. In addition, given that “verbal communication must be employed as the primary means to present [...] visual information to people who are blind” [10], NLIs emerge as a reasonable interactive approach that enables blind persons to access and navigate diagrams.
- Web-based NLIs do not require users to install new hardware or software, since they can be accessed, like any other well-designed website, through the combination of standard Web browsers and assistive technologies such as screen readers. This allows users to forego most of the training time which would be otherwise required to operate a more complex traditional user interface, thus fostering their take-up by the visually impaired population.
- Natural language can express information at different conceptual levels. The knowledge embedded in a diagram can be communicated from low-level facts e.g. the specific value of an individual data point, to high-level, abstract concepts e.g. the general trend described by a time series, thereby preserving, to a certain extent, the functional equivalence of the original diagram regardless of its complexity. This functional equivalence is however limited by the fact that natural language, unlike tactile approaches, is unable to provide direct perceptual access to spatial information [16]. On the other hand, a one-to-one correspondence between the original diagram and its tactile counterpart leads to perceptually cluttered and unusable displays except for very simple diagrams [16]. As a result, most blind persons do not even attempt to read a tactile diagram [7].
- A number of annotation techniques, some of which are discussed in [14], may be employed to compensate for the lack of sight when employing a NLI. These techniques include the ability for users to annotate and bookmark individual graphic objects, high-level summary generation, sequential and hierarchical navigation of graphic objects, clustering of related graphic objects, navigational breadcrumbs, and quick-jumps to salient nodes of the diagram.

However, despite their increasing popularity, research on NLIs is rarely motivated by the goal of accessibility [5]. Existing approaches to accessibility of diagrams supported by natural language are either constrained to providing static, high-level descriptions of the diagram’s contents (see e.g. [4]), or are limited to providing output in natural language, whereas input is given by means of keyboard combinations that need to be learned (see e.g. [6]). Therefore, we have designed AUDiaL as a pure NLI in which both user input and system output are characterized in natural language. In the following, we briefly outline the architecture of AUDiaL and some preliminary evaluation results with blind users.

**Fig. 1.**
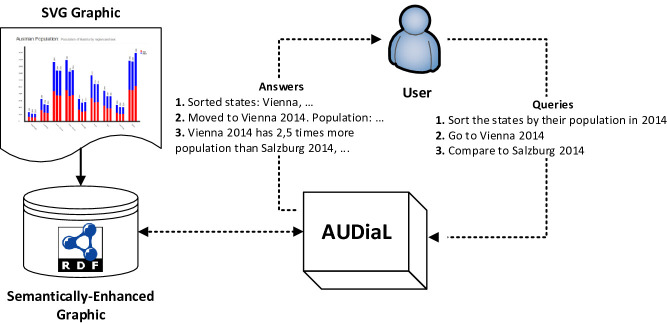
High-level overview of AUDiaL in its problem context. AUDiaL enables (blind) users access information that is visually displayed in statistical charts by means of queries performed in natural language. Example diagram, user queries, and system answers are shown.

## AUDiaL: Accessible Universal Diagrams Through Language

AUDiaL is a Web-based prototype of a natural language interface (NLI) to semantically-enhanced statistical charts in RDF^1^. It was designed with the goal of evaluating whether blind persons find NLIs a usable, effective means of accessing information displayed visually. Figure 1 displays AUDiaL in its problem context. Namely, the developed framework consists of two core independent components:

- ***Semantically-enhanced graphic***: The semantics embedded in a diagram can be characterized at different levels of abstraction by means of formal underpinnings i.e. a knowledge base (KB) on visualization [13]. We have developed a hierarchical set of ontologies for visualization, whose resources may be associated to graphical primitives of a vectorized diagram in SVG format in order to formalize its semantics. This combination of raw SVG graphical data and its associated formal semantics is known as a semantically-enhanced graphic.
- ***AUDiaL prototype***: A Web-based NLI allows blind persons to execute analytical and navigational low-level tasks on a semantically-enhanced graphic. As introduced in [12], we first carried out an analysis on which tasks are commonly undertaken by sighted persons with the support of visualization techniques, and characterized them as formal resources in our hierarchy of ontologies for visualization. AUDiaL allows these tasks to be performed in a non-visual manner by means of queries in natural language. The rest of this paper describes AUDiaL in greater detail and reports some initial evaluation results.

**Fig. 2.**
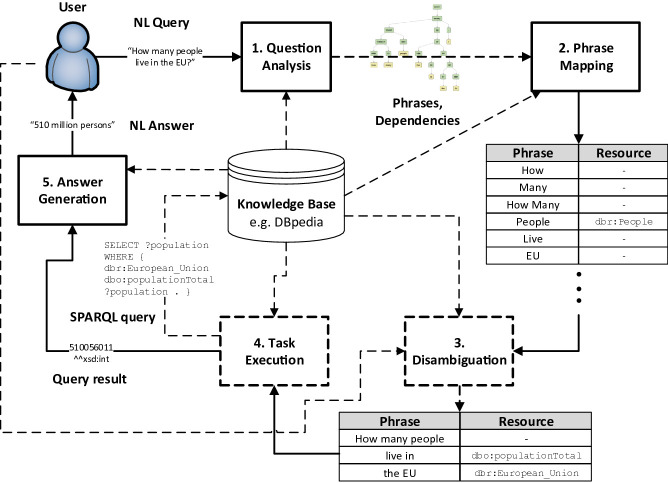
Prototypical process flow of question answering over knowledge bases.

## Natural Language Processing Pipeline

The workflow of a prototypical natural language (NL) to knowledge bases (KB) pipeline is shown in Fig. 2. AUDiaL implements all phases of such a pipeline, where the KB consists of a semantically-enhanced graphic and, optionally, one or more domain ontologies that augment the knowledge of the diagram with external domain knowledge, as follows.

1. ***Question Analysis***: This phase analyses the syntactic features of the input natural language query. The input query is first normalized. Next, a Parse Tree (PT) of the query is generated via an external Stanford CoreNLP Server [11] instance. The PT is then analyzed following several heuristics resulting in a number of Potential Ontology Concepts (POCs). POCs are phrases of the input query that have the potential of being mapped to resources in the KB (e.g. “People” and “EU” in Fig. 2). We generate POCs by extending the approach of FREyA [2]. In addition, we introduce the concept of Cardinal Query Filters (CQFs), which are also generated during this phase and correspond to phrases of the query that modify the selection of triples retrieved from the KB before executing a task or the triples that are output as a result of executing a task (e.g. “more than 1000” or “approximately 20”).
2. ***Phrase Mapping***: This phase aims to map each of the query’s phrases found during the previous phase to zero or more resources of the KB. For example, the phrase “People” could be mapped to a literal resource, for example "People⌃⌃xsd:string" underpinning a label’s text in a semantically-enhanced diagram through a datatype property occurrence. This phase not only searches for query phrases as-is in the KB, but also lemmatized versions and synonymic phrases thereof.
3. ***Disambiguation***: The disambiguation phase resolves any remaining POCs, QCFs, and disambiguates between KB resources that have been mapped to the same query phrase during the previous phase of the pipeline. An automatic consolidation phase takes place first, which aims to automatically resolve any unresolved and ambiguous phrases. All remaining unresolved elements are settled by explicitly asking the user in mapping and disambiguation dialogues following the approach described in [3] which we have extended to include QCF resolution. An example mapping dialogue is shown in Fig. 6 along with further information about this phase.
4. ***Task Execution***: This phase is in charge of executing the corresponding task stemming from a consolidated user query. First, the resources corresponding to the task(s) to be executed are identified. If no task is found, it defaults to a filtering task in which all elements in the graphic that match the user’s query are simply listed. Next, a number of KB triples that match the resources and CQFs of the consolidated user query are retrieved. Lastly, the chosen analytical task is executed on the matching triples. For example, some of the tasks currently recognized include retrieving an average of values, determining the trend of values with respect to a metric axis, clustering graphic objects according to some of their attributes, or jumping between graphic objects. The complete list of implemented low-level tasks can be consulted in [12].
5. ***Answer Generation***: Lastly, the task result is expressed in a suitable manner, depending on the task, in natural language and dynamically embedded into AUDiaL’s accessible Web interface. The user may now input a new query, and the processing pipeline will be executed again from the beginning.

Besides retrieving knowledge from the graphic and domain ontologies, AUDiaL additionally supports user-specific annotation of graphic elements in order to aid users with navigating complex graphics more efficiently by bookmarking elements, selecting home nodes during navigation, or simply adding customized information to that already contained in the diagram. Moreover, a high-level summary of the graphic as a whole may be requested at any time [14]. These techniques aim to compensate for the lack of sight of AUDiaL’s user base. For example, bookmarking methods were implemented with the goal of preventing the overloading of the users’ working memory when navigating complex diagrams.

## Evaluation

An initial evaluation of AUDiaL was carried out with 9 visually impaired participants (5 females, 4 males; 7 fully blind, 2 near blind; average age $\mu = 24.11$, standard deviation $\sigma = 12.17$), all of whom had more than 5 years Web browsing experience and who were familiar with the concepts of bar and line charts. Most of them (89%) reported that their preferred means for accessing diagrams was a textual or tabular version thereof, with a single person reporting a preference for tactile graphics.

A statistical difference-making experiment [18] was designed, as follows. Two diagrams (a stacked bar chart and a simultaneous combination of a bar chart and a line chart sharing the same metric space) of similar complexity (depicting the equivalent of around 80 tabular values each) were semantically enhanced with Semantic Annotator for Inkscape (SAI) [15] and uploaded to a running instance of AUDiaL. Participants were provided user accounts and asked to solve eight tasks of varying difficulty on both diagrams. Around half of the participants were asked to answer the first batch of tasks using AUDiaL, and the second batch of questions using a tabular or tactile version of the diagram according to their individual preference. The other half of the participants were asked to employ the traditional alternative first, followed by AUDiaL. This process, known as a complete counterbalanced, repeated measures, task-based evaluation design, enables us to halve the number of participants needed while minimizing potentially harmful sequence effects (e.g. participant fatigue) in the evaluation [2].

Tasks included initial simple questions that could be answered by consulting the high-level summary of the diagram e.g. “How many bars are there in the chart?”, meant to get participants accustomed to using the prototype; tasks of intermediate difficulty that could not be solved by asking a single question and introduced mapping and disambiguation dialogues e.g. “Write the two Austrian regions having the maximum and minimum population in 2004, respectively”, to complex questions that required participants to acquire a mental model of the diagram as a whole e.g. “Which regions increased in population between 2004 and 2014?”. Participants were not trained in using the prototype, being simply instructed to input free-formed questions in the only text field of the user interface. Each evaluation session lasted for approximately 150 min, with two intermediate breaks. System logs were collected in order to determine the time it took participants to solve (or withdraw) each task. Moreover, the efficiency in solving each task in a scale from 0 (solved it with ease) to 2 (task failed) was gathered. Participants were also asked to answer two complementary user satisfaction surveys.

**Fig. 3.**
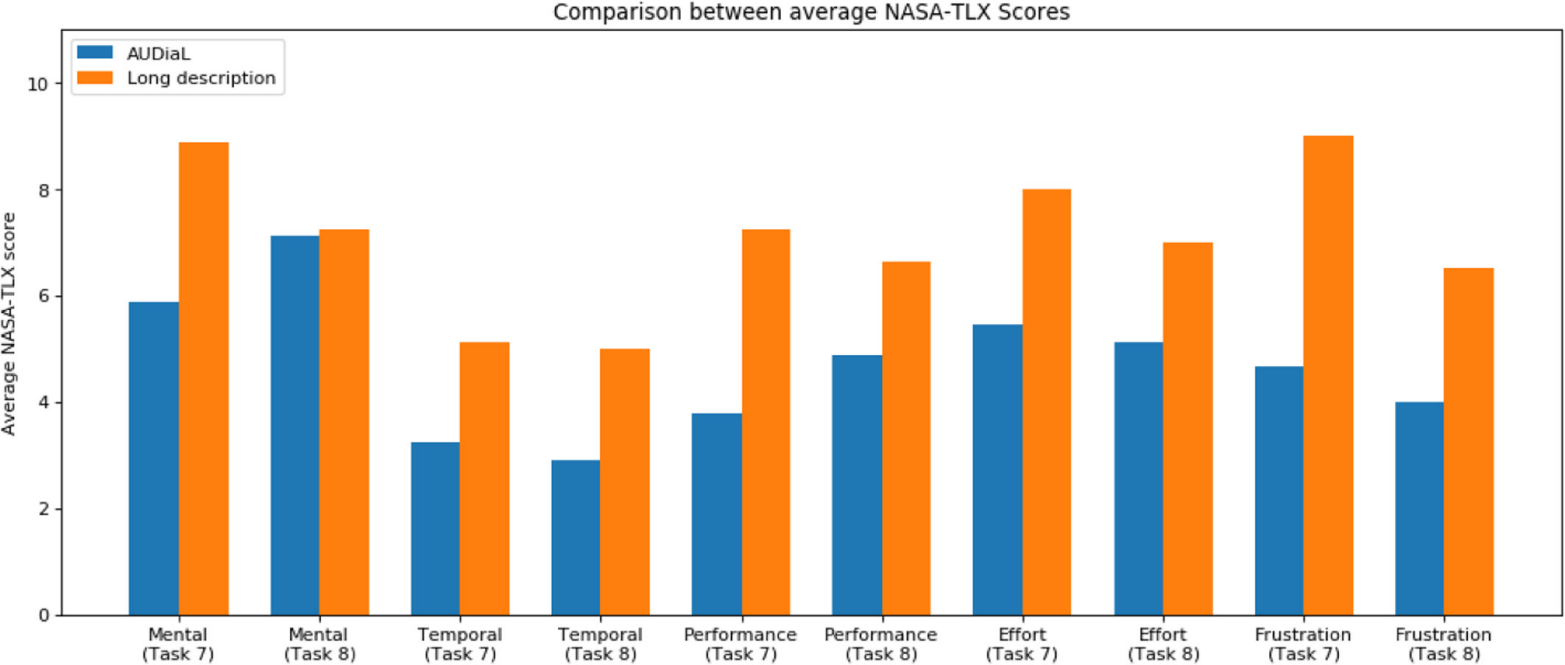
Comparison of average NASA-TLX scores.

**Fig. 4.**
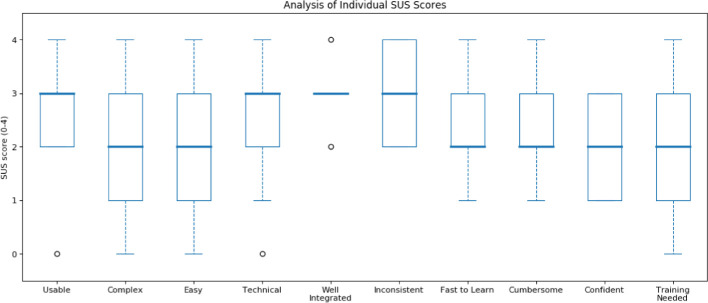
Analysis of individual SUS scores obtained by AUDiaL.Analysis of individual SUS scores obtained by AUDiaL.

Given that the number of participants is insufficient to derive a statistically significant measure of the differences in task efficiency and efficacy between using AUDiaL as opposed to other methods, in the following we lay our focus on reporting the user satisfaction metrics we have collected. After having completed the two hardest tasks (task 7 and 8) with either AUDiaL or by consulting the diagram’s alternative long description, users were asked 5 feedback questions in order to compute the NASA Task Load Index (NASA-TLX, [9]) scores for each task. This index is a widely used assessment tool that rates user perceived workload to assess a system’s effectiveness. Results, as depicted in Fig. 3, show that, on average, users rate tasks performed on AUDiaL as less mentally demanding, faster to solve, and less frustrating than the same tasks being solved with support of a long description of the diagram.

**Fig. 5.**
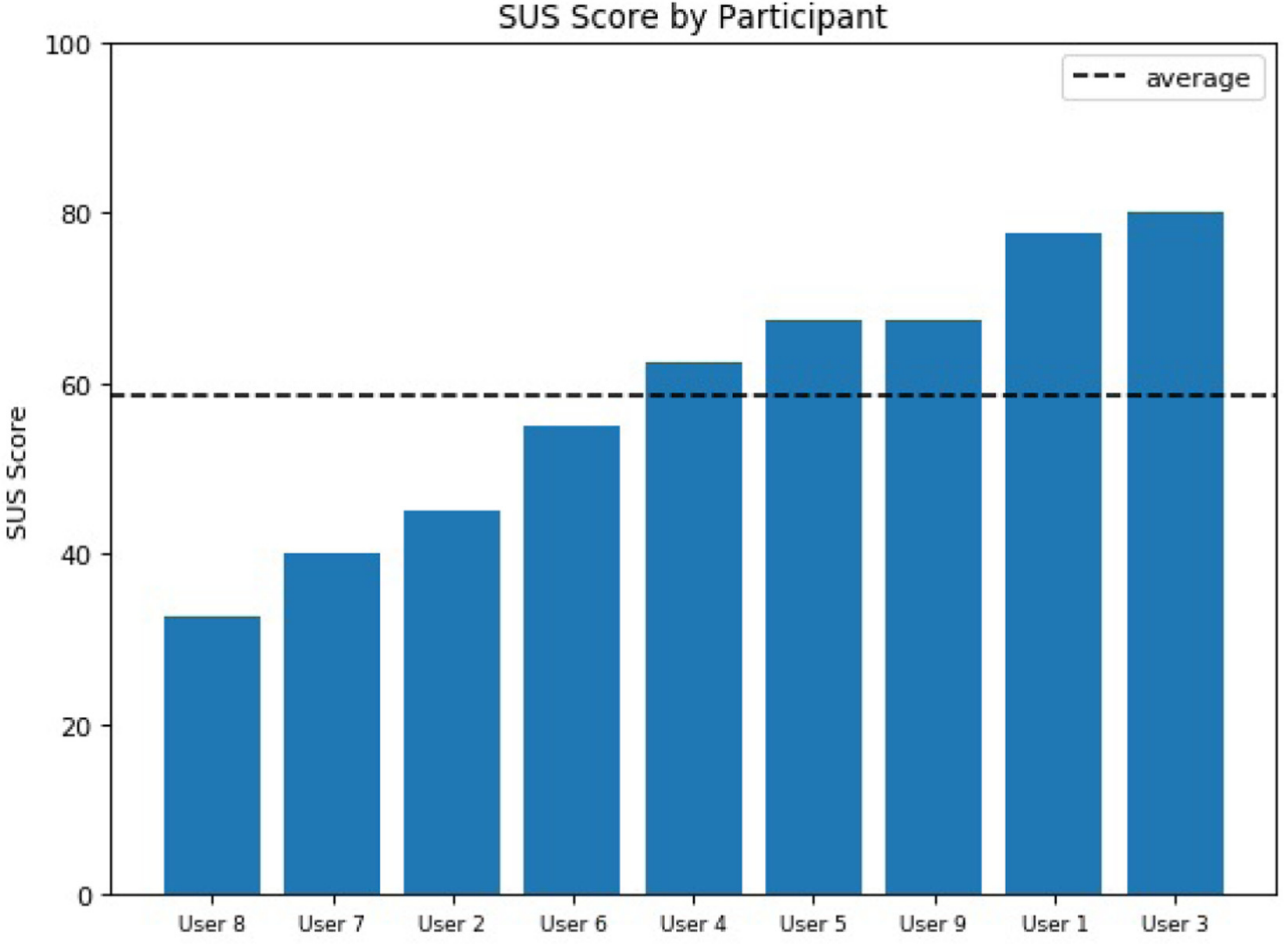
SUS scores by participant.

**Fig. 6.**
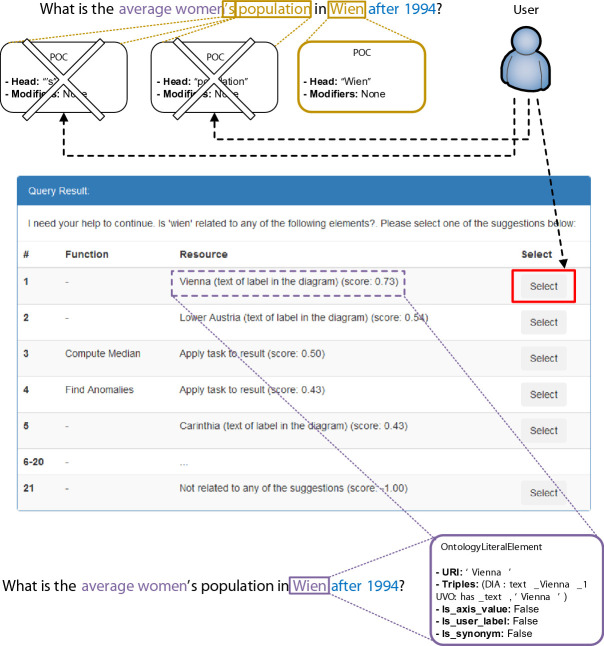
Example of an (adapted) mapping dialogue. The user should select the resource on the 1st row (the literal *Vienna*) as the resource that the POC corresponding to the phrase “Wien” should be mapped to, resulting in the resolution of the unknown phrase (“Wien”) to a specific resource of the underlying semantically-enhanced graphic (*Vienna*). The remaining two POCs were previously discarded by mapping them to *None* elements (last row of the mapping dialog). This process results in a consolidated user query that may now be resolved in the task execution phase of the processing pipeline. The bottom of the figure shows a consolidated query consisting of three ontology concepts (“average”, mapped to an analytical task; and “women” and “Wien”, mapped to literals corresponding to labels in the diagram), and a CQF (“after 1994”) which will filter out the output triples having objects less than or equal to 1994 before generating the final answer.

The last part of the evaluation had participants answer the System Usability Scale (SUS) questionnaire (the *de facto* standard satisfaction measure of system usability evaluation in industry [1]) about their experiences with AUDiaL. SUS has been shown to produce the most reliable results among all sample sizes when compared to four other website usability questionnaires. Moreover, it can be used on small sample sizes with reliable results, with a reported accuracy of 75% with a sample size of 8 [17]. The results, shown in Fig. 4 and Fig. 5, exhibit that participants had somewhat mixed subjective impressions of AUDiaL. On the one hand, users found it, for the most part, usable and consistent. However, the presence of many dialogues (Fig. 6) that had to be resolved before an answer to some queries could be computed resulted on some users finding the prototype complex to use. This resulted in most participants resorting to a strategy of asking simple, short questions that they felt were less likely to provoke system prompts. The obtained average SUS score of 58.61 is an acceptable result. However, as seen in Fig. 5, scores greatly varied between individual participants. When inquired individually, users that gave the lowers scores reported a very low interest in statistics and mathematics in general. For example, the participant who gave the lowest score mentioned that she “hated maths” and initially thought the session was about tactile geographical maps. Another participant who gave AUDiaL a low usability score reported that he felt he was “just crunching numbers”. However, he conceded that AUDiaL was “easier and much faster” than using a table or a tactile graphic. Another participant reported that even she though the system was “interesting and useful for blind people” she “would rather read a table” because she was already used to her screen reader’s shortcuts when navigating HTML tables. This was a common motif among participants, who often requested for a list of available commands or queries understood by the system. Another common complaint was that the suggestions given by the mapping and disambiguation dialogues were not clear, especially when inputting long, complex queries.

On the other hand, participants who gave the highest score had previously attended courses on statistics and were generally more engaged and interested in the tasks performed during the evaluation, declaring that the prototype was “really fun” and that they would likely use such a system in their day-to-day lives. A participant reported that AUDiaL made the diagram “easier to understand” compared to the long description alternative, since she would always “get stuck” on the table. Another one said that AUDiaL was “very interesting” and that she enjoyed the interactivity it provided as opposed to the static nature of the table. While solving the given tasks on the tabular alternative, she commented “this is not so much fun” and that she would rather go back to using the dialogue prototype if possible. A different user said “I wish I had more time to get to know this system, I am sure I could do better”.

## Conclusions and Further Work

In conclusion, the evaluation results outlined in this paper show that NLIs display promising results in allowing blind persons to access information displayed visually in statistical charts in an autonomous and natural manner. Blind persons with a previous interest in data analysis and statistics found NLIs an engaging and effective means of accessing diagrams preferable to their traditional counterparts. On the other hand, our NLI prototype failed to provoke an interest in such matters in the case of participants already apathetic about them. These results suggest that such an approach may be of special interest in educational settings, where blind and sighted students could engage together in the same problems supported by diagrams.

The implemented NLI still presents much room for improvement, especially regarding the disambiguation phase of the NLP pipeline (Fig. 2). In addition, time constrains during evaluation sessions prevented us from evaluating whether the techniques that aim to compensate for the lack of sight when navigating complex diagrams (e.g. node annotation) enable blind persons to use the NLI more efficiently. In the future we intend to evaluate these aspects as well as an improved version of AUDiaL.

## Footnotes

1 We are currently working on guidelines for the authoring of semantically-enhanced diagrams in RDF. For a general introduction to RDF, the reader is referred to the RDF primer, available at https://www.w3.org/TR/rdf11-primer/.
